# An Investigation of Size Distribution and Calcium Signaling in Human Platelets

**DOI:** 10.7759/cureus.59547

**Published:** 2024-05-02

**Authors:** Koushik Biswas, Susheel N Chaurasia, Debabrata Dash

**Affiliations:** 1 Biochemistry, All India Institute of Medical Sciences, Raebareli, Raebareli, IND; 2 Biochemistry, Institute of Medical Sciences, Banaras Hindu University, Varanasi, IND

**Keywords:** calcium ionophore a23187, talin, platelet size, platelet-derived microparticles, platelet

## Abstract

Background

Platelets are thin disc-shaped blood cells that play a major role in hemostasis, maintenance of vascular integrity, and blood coagulation. Large platelets are more reactive and seen in patients with cardiovascular disease. This study aims to analyze the changes in platelet size of ex vivo activated platelets which phenotypically simulates that of a patient at risk of cardiovascular disease and elucidate the calcium signaling pathway responsible for this change.

Methodology

Platelets were isolated from adult human blood by differential centrifugation. Calcium was mobilized into platelets by treatment with calcium ionophore A23187 in the presence of Ca^2+^. Platelet size distribution was analyzed using Coulter Counter Multisizer 4. The following signaling parameters were studied: intracellular Ca^2+^ measurement (using Fura-2/AM by fluorescence spectrophotometry), Ca^2+^-dependent thiol protease calpain assay (using fluorogenic substrate *t*-butoxycarbonyl-Leu-metchloromethylcoumarin in fluorescence microplate reader), platelet-derived microparticles (using FACS Calibur flow cytometry), and cytoskeletal protein talin expression (by western immunoblotting).

Results

When adult platelets were treated with A23187 and Ca^2+^, two subcellular populations (<2 µm and between 2-4 µm) were noted. The mean size of the second cell population was significantly higher than that of resting platelets (2.94 ± 0.13 µm vs. 2.82 ± 0.15 µm, t = 4.605, p = 0.00). A23187 treatment led to elevated intracellular Ca^2+^, release of platelet-derived microparticles, increase in calpain activity, and cytoskeletal talin degradation. These events were inhibited by calpeptin (a specific calpain inhibitor).

Conclusions

Elevated calcium caused talin degradation by calpain activity. Breakdown of this cytoskeletal protein leads to relative swelling of cells reflected by the increase in platelet size.

## Introduction

Platelets are thin disc-shaped blood cells that play a major role in hemostasis, maintenance of vascular integrity, and blood coagulation [1]. The normal platelet size ranges from 2 to 4 µm and the normal count ranges from 150,000 to 400,000/µL. These cells have a lifespan of about 8-10 days in circulation [1,2].

Platelet size reflects the platelet activity. Platelets with large sizes are enzymatically and metabolically more active than those with a smaller size and have been known to produce more thromboxane A2 [3,4]. The mean platelet volume (MPV) depends on the platelet size and is considered to be a marker of platelet function and activation. Changes in platelet production or platelet stimulation alter the MPV [5]. Elevated MPV is seen in those at risk of cardiovascular diseases [6].

Patients with atherosclerosis are reported to have active circulating platelets compared to resting platelets in healthy individuals [7]. These activated platelets have elevated intracellular Ca^2+^ levels [8]. This study aims to analyze the changes in platelet size of ex vivo activated platelets which phenotypically simulates the activated platelets of a cardiovascular disease patient and elucidate the signaling pathway responsible for this change.

## Materials and methods

This quasi-experimental study was conducted in the Department of Biochemistry, Institute of Medical Sciences, Banaras Hindu University, Varanasi, India over two years. Human volunteers aged 18-59 years arriving at the Blood Bank of Sir Sunderlal Hospital located within the university for voluntary blood donation were enrolled in the study based on a convenience sampling technique. A venous blood sample was collected after obtaining written informed consent, strictly as per recommendations and as approved by the Institutional Ethical Committee (approval number: Dean/2012-13/100).

Platelet preparation

Platelets were isolated by differential centrifugation from fresh human blood donated by healthy volunteers, as already described [9]. All platelet preparation steps were performed under aseptic conditions while taking precautions to maintain the platelets in a resting condition.

Platelet size estimation

Washed platelets were preincubated with Ca^2+^ (1 mM) for two minutes followed by treatment with A23187 (1 µm) for 15 minutes. Dimethylsulfoxide (DMSO) was added as the vehicle in control samples. The size and distribution of platelets in both groups were analyzed after 15 minutes of treatment using Beckman Coulter Multisizer 4. An internal quality control check was performed daily before running samples.

Measurement of intracellular free calcium

Intracellular free calcium was measured in Fura 2-acetoxymethyl ester (Fura-2 AM)-stained platelets, as already described [9], and calibrated as per the derivation of Grynkiewicz et al. [10].

Calpain activity assay

Intracellular calpain activity was measured, as described previously [11]. The following three different samples were taken: (i) washed platelets, (ii) washed platelets treated with calcium ionophore A23187 (1 µM) and Ca^2+^ (1 mM), and (iii) washed platelets preincubated with calpeptin and then treated with A23187 (1 µM) and Ca^2+^(1 mM). These samples were incubated in 96-well microplates for 15 minutes and then loaded with the calpain substrate, t-butoxy carbonyl-Leu-metchloromethylcoumarin (10 µM). After 30 minutes of incubation, cellular fluorescence was estimated using a fluorescence microplate reader (BioTek model FL×800) at 37°C using 351 nm excitation and 430 nm emission filters [12].

Analysis of platelet-derived microparticles

Washed platelets were treated either with A23187 (1 μM) or vehicle (DMSO) for 20 minutes in the presence of CaCl_2_ (1 mM), and centrifuged at 3,000 rpm for five minutes. Supernatants containing platelet-derived microparticles were incubated with 5 μL PE-labeled annexin V in the presence of an annexin-binding buffer containing calcium for 30 minutes in the dark at room temperature. Samples were then analyzed by flow cytometry (FACSCalibur, BD Biosciences). An appropriate gate was drawn to encompass and differentiate platelet-derived microparticles from platelets. Forward and side scatter voltages were set at E00 and 350, respectively, with a threshold of 52 V. Fluorescence data were collected using four-quadrant logarithmic amplification for 10,000 events from each sample within the gate and analyzed using Cell-Quest Pro Software [12].

Electrophoresis and immunoblotting studies

After treatment, platelets were lysed with Laemmli sample lysis buffer, boiled, and the proteins obtained were separated on 10% polyacrylamide gels using sodium dodecyl sulfate-polyacrylamide gel electrophoresis.

After separation on 10% polyacrylamide gel, platelet proteins were electrotransferred onto a polyvinylidene fluoride membrane by applying 0.8 mA of current per cm^2^ of gel for one hour and 45 minutes using the TE77 PWR semidry system (GE Healthcare). Membranes were blocked with 5% non-fat milk in TBST buffer (10 mM Tris-HCl, 150 mM NaCl, pH 8.0 containing 0.05% Tween-20) for one hour at room temperature. Blots were then incubated overnight with primary antibody (anti-talin) diluted in 5% bovine serum albumin (BSA) in TBST (1 µg/mL) at 4°C. On the next day, blots were washed three times with TBST buffer for five minutes each time. Blots were incubated with anti-mouse IgG horseradish peroxidase-conjugated secondary antibody (diluted 1:40,000 with TBST containing 5% BSA) for one hour. Blots were washed thrice with TBST, and protein was detected using an enhanced chemiluminescence detection kit. Images were acquired on a multispectral imaging system (Biospectrum 800 Imaging System; UVP; Medispec, India) and quantified using VisionWorks LS software (UVP).

Sample size

We included blood samples from 40 healthy volunteers in our study based on the inclusion and exclusion criteria. As the washed platelets of each person were divided into two parts and given separate treatments (A23187 or DMSO), we assumed that age, sex, and other confounding factors were nullified.

Inclusion and exclusion criteria

We included healthy volunteers aged 18-59 years. We excluded known cases of hypertension, diabetes mellitus, chronic kidney disease, or any other chronic disease. Those with a history of cerebrovascular accident, malignancy, or tuberculosis were also excluded. Patients with any acute episode of illness in the last year were excluded. Those presently taking any medication were excluded. Those with a history of substance abuse in the present or past were excluded. Individuals who refused to provide informed consent for the study were excluded.

Statistical analysis

All data were checked for normality using the Kolmogorov-Smirnov test. The parametric data were presented as mean ± standard deviations, while the non-parametric data were presented as median and interquartile range. A paired t-test was used to compare the difference in mean between washed platelets receiving two different types of treatment (A23187 and DMSO). A p-value <0.05 was considered significant. Data were analyzed using SPSS version 25 (IBM Corp., Armonk, NY, USA).

## Results

We separated washed platelets of healthy adult volunteers into two parts. One part was treated with A23187 (1 µM) in the presence of Ca^2+^ (1 mM). The other part was treated with DMSO (vehicle) and Ca^2+^ (1 mM). After 15 minutes of incubation at room temperature, both were resuspended in isotone separately, and the variation in platelet size distribution was analyzed using Beckman Coulter Multisizer 4.

**Figure 1:**
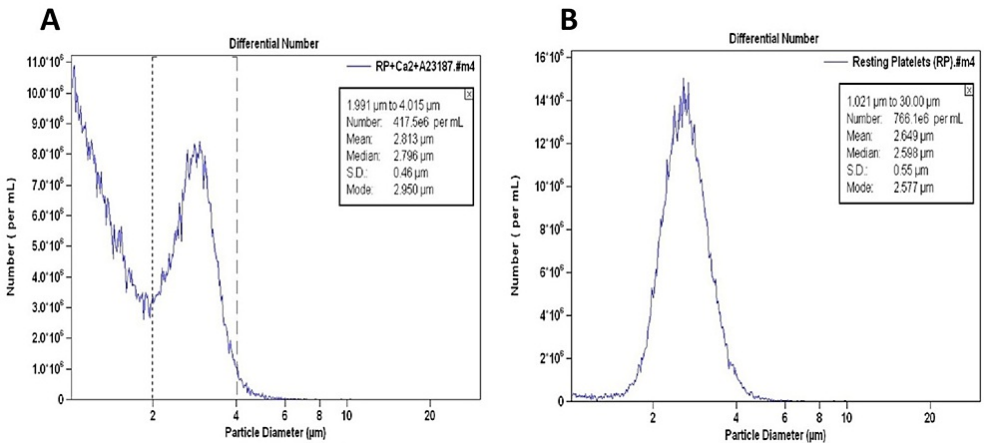
Size distribution of washed platelets from healthy adult volunteers (A) treated with Ca2+ (1 mM) and A23187 (B) treated with Ca2+ (1 mM) and DMSO (vehicle).

We observed two subcellular populations in the washed platelets treated with A23187 and Ca^2+^: one less than 2 µm and the other from 2-4 µm (Figure 1A). However, in the washed platelets treated with DMSO (vehicle) and Ca^2+^, only one type of cell population, the majority of which measured 2-4 µm, was noted (Figure 1B).

**Figure 2:**
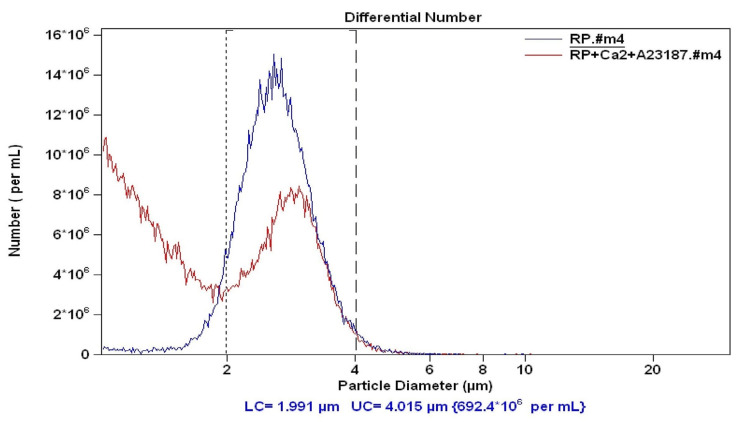
Washed platelets from healthy adult volunteers treated with Ca2+ (1 mM) and A23187 (red curve) compared to washed platelets treated with Ca2+ (1 mM) and vehicle DMSO (blue curve).

On comparing the mean size of the subcellular population of 2-4 µm size range in both A23187 and DMSO-treated washed platelets, we observed that the mean size was more in the A23187-treated compared to the DMSO-treated subcellular population. This is seen as a right shift in the red curve (depicting A23187 treated cells) compared to the blue curve (depicting DMSO treated cells) in Figure 2. A paired t-test revealed that this increase in the mean size of the subcellular population (2-4 µm range) on A23187 treatment was statistically significant (2.94 ± 0.13 µm vs. 2.82 ± 0.15 µm, t = 4.605, p = 0.00).

We also observed that treatment of platelets with A23187 and Ca^2+^ led to an increase in the number of subcellular populations of less than 2 µm size range. Subcellular populations in this size range (<2 µm) were not noted in such a high number in the resting platelets.

**Figure 3:**
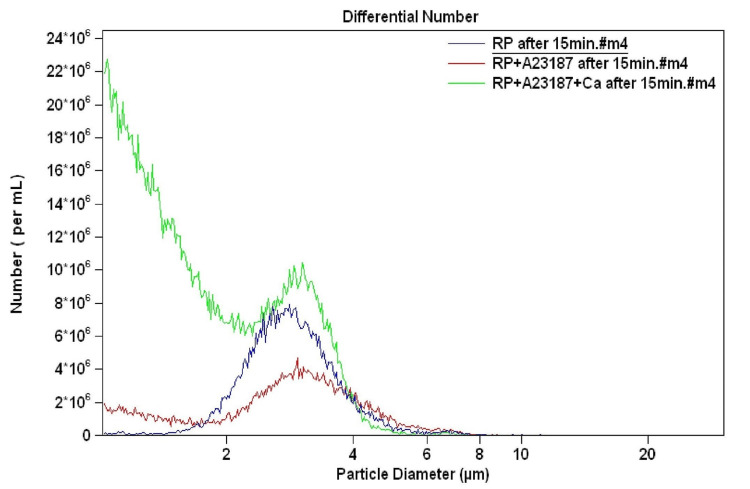
Washed platelets from healthy adult volunteers treated with A23187 in the presence (green curve) and absence (red curve) of Ca2+ (1 mM).

We attempted to understand if Ca^2+^ had any effect on the size distribution in ionophore-treated platelets. Resting platelets were treated with A23187 (1 µM) in the presence or absence of Ca^2+^ (1 mM) and incubated for 15 minutes at room temperature. We observed that A23187 alone (in the absence of Ca^2+^) did not generate the subcellular population of <2 µm size range, but it still increased the mean size of the subcellular population of the 2-4 µm range. Hence, it was confirmed that the increase in the size of the subcellular population (2-4 µm size range) was due to A23187 and not Ca^2+^ (Figure 3).

**Figure 4:**
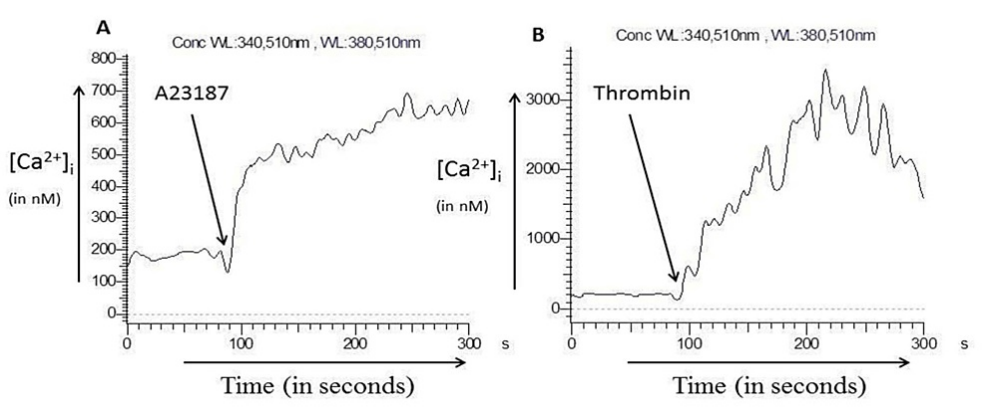
Intracellular Ca2+ measurement. (A) A23187-induced rise in intracellular Ca^2+^ in platelets. A23187 (1 µM) was added to Fura-2-loaded platelets in the presence of Ca^2+^ (1 mM). (B) Thrombin-induced rise in intracellular Ca^2+^ in platelets. Thrombin (1 U) was added to Fura-2-loaded platelets in the presence of Ca^2+^ (1 mM).

As cytosolic Ca^2+^ is an important component of cell signaling, we next checked the effect of ionophore A23187 on intracellular Ca^2+^ concentration, [Ca^2+^]i. A23187 (1 µM) and thrombin (1 U) (control) were added to Fura-2 loaded platelets in separate experimental sets in the presence of Ca^2+^ (1 mM). We observed that A23187 resulted in an initial rise in [Ca^2+^]i from basal level (100-200 nM) to a higher level (500-700 nM), following which its level remained in a steady state (plateau shape of the curve). Thrombin (control) resulted in an initial rise and a slight fall in intracellular Ca^2+^ (Figure 4).

**Figure 5:**
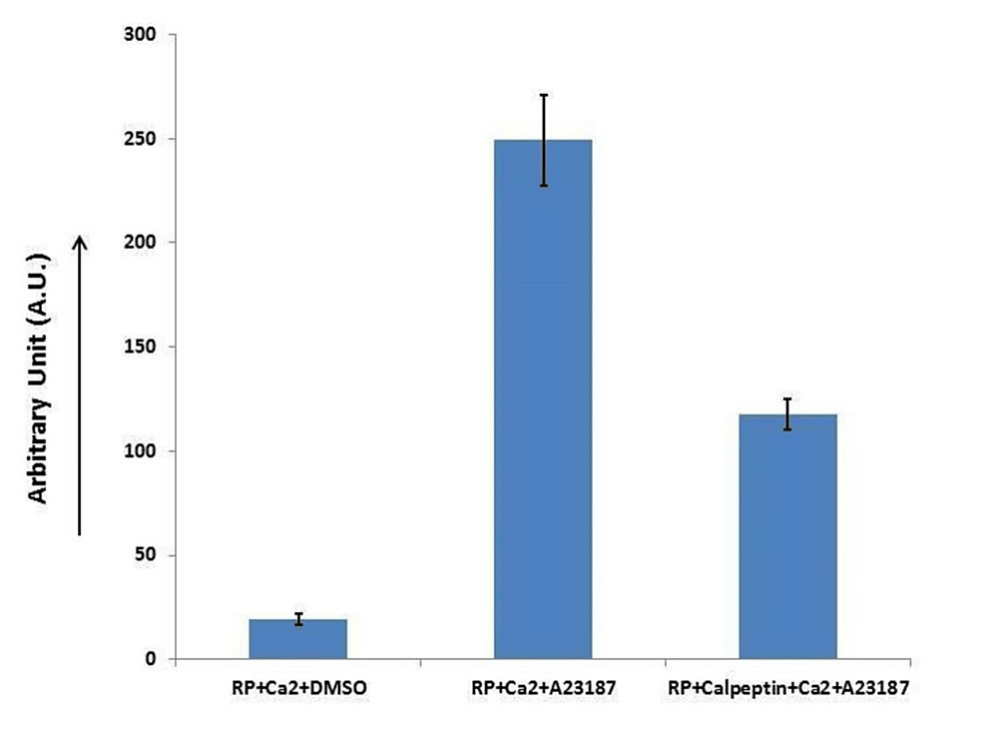
Calpeptin reduces A23187 calpain activity in platelets. Data presented as mean ± standard deviation of five independent experiments.

Next, we checked if an A23187-induced rise in platelet intracellular calcium concentration enhances calpain activity in platelets. We observed that ionophore A23187 (1 µM) in the presence of Ca^2+^ (1 mM) induced a 13 to 15-fold rise in platelet calpain activity. However, when cells were preincubated with calpeptin (80 µM), a specific calpain inhibitor, calpain activity was found to be reduced by about twofold, thus implicating calpain activation in A23187-treated platelets (Figure 5).

**Figure 6:**
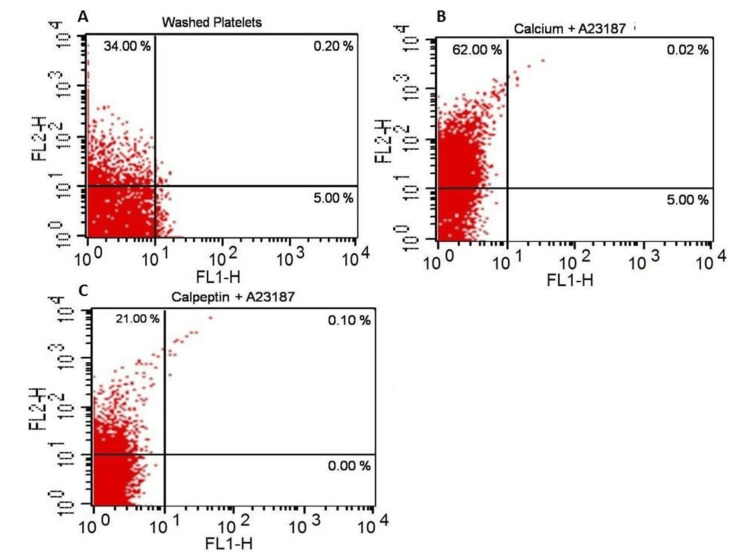
A23187-induced rise in platelet-derived microparticles. Cells treated, as mentioned, were analyzed with a flow cytometer after gating of the microparticle region distinct from platelets. Dot plots representing FL-1 and FL-2 fluorescence of 10,000 events. The number within parenthesis in each quadrant denotes the percentage of total gated events in respective quadrants.

We analyzed the A23187-induced shedding of microparticles from platelets by flow cytometry using an annexin V antibody in an FL-2 channel. We observed a higher number of platelet-derived microparticles in A23187 (1 μM) and Ca^2+^ (1 mM)-treated washed platelet samples. This decreased when the sample was pretreated with calpeptin (Figure 6). This observation confirmed that calpeptin inhibits calcium ionophore-induced generation of microparticles from platelets.

**Figure 7:**
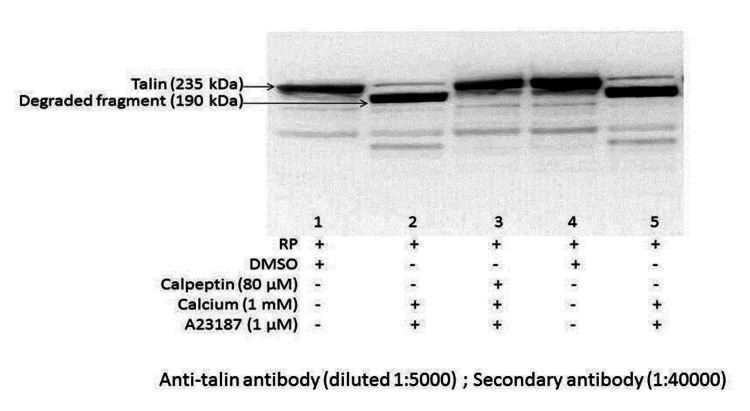
Western blotting showing the effect of ionophore A23187 on platelet membrane protein degradation.

We conducted electrophoresis followed by western blotting of platelet proteins. We observed the complete disappearance of the platelet cytoskeletal protein talin (Mr 235 kDa), with the appearance of a cleaved peptide at 190 kDa, when washed platelets were treated with A23187 (1 µM). Preincubation with calpeptin, a calpain inhibitor, completely prevented A23187-mediated proteolysis of talin (Figure 7).

## Discussion

In this study, we drew blood from healthy volunteers, separated platelets, and treated it in vitro with calcium ionophore (A23187) and Ca^2+^ to activate it so that they simulate the activated platelets of patients with cardiovascular diseases. We then observed the changes in platelet size distribution and attempted to correlate the intracellular signaling mechanisms leading to changes in platelet size distribution. Platelet size distribution, as analyzed by Multisizer 4 using the Coulter principle, showed that the majority of the washed platelets (over 85%) measured 2-4 µm. This correlates with the already established literature [1].

A23187 transports divalent cations (e.g., Ca^2+^, Mg^2+^) across the cell membrane into the cytoplasm of the platelet and can also release Ca^2+^ from intracellular storage sites. An increase in platelet cytosolic Ca^2+^ from extrinsic or endogenous sources after exposure to A23187 is reported to stimulate shape change, aggregation, and secretion by triggering the contractile mechanism of the cell [13]. In our study, we observed that when platelets were treated with Ca^2+^ and A23187, platelets were divided into two subcellular populations (Figure 1A). One which was less than 1 µm size corresponded in size with the platelet-derived microparticles. We further confirmed this subcellular fragment population to be platelet-derived microparticles by flow cytometry (Figure 6). Kailashiya [14] also reported the size of the platelet-derived microparticles to be 0.1-1 µm.

The second subcellular population observed was 2-4 µm in size, and the mean size of this population was significantly higher compared to the mean size of resting platelets (Figure 2). This cell population represented the larger platelets in patients with cardiovascular disease. Guthikonda et al. [15] had previously reported that in patients with stable cardiovascular disease, larger platelets were more reactive and more prone to aggregation.

We then wanted to assess if the increase in the mean size of platelets was due to A23187 or extracellular Ca^2+^. We observed that even when the washed platelets were treated with A23187 in the absence of extracellular Ca^2+^, their size increased. However, in this case, the platelet-derived microparticles were not generated in such a high proportion (Figure 3). When we checked the effect of ionophore A23187 on intracellular Ca^2+^ concentration, we observed that A23187 treatment led to an increase in the intracellular Ca^2+^ concentration (Figure 4). These experiments elucidated that the increase in the platelet size was linked to an increase in the intracellular Ca^2+^ concentration. Larger platelets are hypothesized to be a more reactive subpopulation that aggregates faster upon stimulation [16].

We checked the downstream changes associated with elevated intracellular calcium. Figure 5 depicts that platelet calpain activity increased 13 to 15-fold in A23187 and Ca^2+^ treatment. However, this was prevented when platelets were pretreated with calpeptin. This highlights that elevated intracellular Ca^2+^ is responsible for increasing the protease calpain activity. Miyoshi et al. [17] reported that tert-butyl peroxide induced a sustained increase in rat hepatocytes, which increased calpain µ activity and resulted in blebbing of the hepatocyte cell membrane. This phenomenon was inhibited by calpeptin.

The platelet cytoskeleton consists of actin, spectrin, and tubulin. This cytoskeleton provides support for the maintenance of the discoid shape and integrity when the platelet is exposed to shear during circulation. It can also rapidly remodel upon activation and allow the platelets to promptly respond in case of vascular damage [1]. Talin is a cytoplasmic protein (Mr 235 kDa) that links the actin cytoskeleton and the extracellular matrix [18]. Talin interacts with the cytoplasmic protein vinculin, [19,20] which can further associate with α-actinin, the actin cross-linking protein [21,22]. In our study, we observed that A23187 treatment led to the complete disappearance of the platelet cytoskeletal protein talin (Mr 235 kDa), with the appearance of a cleaved peptide at 190 kDa. This breakdown was prevented by calpeptin pretreatment (Figure 7). We summarize that activated platelets have increased intracellular calcium levels, which causes downstream signaling such as increased calpain activity, talin breakdown, and platelet-derived microparticle generation. The breakdown of talin disrupted the organized links between the actin cytoskeleton and the extracellular matrix of platelets, resulting in a relative increase in platelet size. The main advantage of our study was that the washed platelet of each individual was divided into two parts and given separate treatments (A23187 or DMSO), which nullified the effect of age, sex, and any other confounding factors.

Our study is not without limitations. The main limitation was that this was an ex vivo study. We separated platelets from whole blood and then activated them ex vivo by ionophore A23187. This may not completely resemble physiologically activated platelets seen in a cardiovascular disease patient. We suggest future researchers separate large platelets of cardiovascular patients by differential centrifugation and then carry out the experiments we have performed.

## Conclusions

We observed that the majority of human platelets from healthy adult volunteers were in the 2-4 µm size range. When treated with ionophore A23187 and Ca^2+^ the platelets divided into subcellular two populations. The first population of less than 1 µm in size corresponded with platelet-derived microparticles. The second population of 2-4 µm in size had a statistically higher mean size than the resting platelets. A23187 treatment resulted in the release of Ca^2+^ from the intracellular stores, thereby increasing intracellular Ca^2+^ levels. Elevated intracellular Ca^2+^ levels increased calpain activity and led to the breakdown of platelet cytoskeletal protein talin. Disruption of the organized cytoskeletal structure of platelets led to a relative increase in the size of the platelets. Calpeptin pretreatment prevented the rise of intracellular Ca^2+^, calpain activation, and talin breakdown.
